# Human Milk Oligosaccharide Supplementation Affects Intestinal Barrier Function and Microbial Composition in the Gastrointestinal Tract of Young Sprague Dawley Rats

**DOI:** 10.3390/nu12051532

**Published:** 2020-05-25

**Authors:** Faye Chleilat, Teja Klancic, Kyle Ma, Alana Schick, Jodi E. Nettleton, Raylene A. Reimer

**Affiliations:** 1 Faculty of Kinesiology, University of Calgary, 2500 University Drive NW, Calgary, AB T2N 1N4, Canada; fatima.chleilat1@ucalgary.ca (F.C.); teja.klancic@ucalgary.ca (T.K.); kma225@uwo.ca (K.M.); jenettle@ucalgary.ca (J.E.N.); 2 International Microbiome Centre, Cumming School of Medicine, University of Calgary, 3330 Hospital Drive NW, Calgary, AB T2N 4N1, Canada; a.schick@ucalgary.ca; 3 Department of Biochemistry & Molecular Biology, Cumming School of Medicine, University of Calgary, 3330 Hospital Drive NW, Calgary, AB T2N 4N1, Canada

**Keywords:** human milk oligosaccharides, 2′-*O*-fucosyllactose, 3′sialyllactose, gut microbiota, intestinal permeability, inflammation

## Abstract

Human milk oligosaccharides (HMOs) are chief maternal milk constituents that feed the intestinal microbiota and drive maturation of the infant gut. Our objective was to determine whether supplementing individual HMOs to a weanling diet alters growth and gut health in rats. Healthy three-week-old Sprague Dawley rat pups were randomized to control, 2′-*O*-fucosyllactose (2′FL)- and 3′sialyllactose (3′SL)-fortified diets alone or in combination at physiological doses for eight weeks. Body composition, intestinal permeability, serum cytokines, fecal microbiota composition, and messenger RNA (mRNA) expression in the gastrointestinal tract were assessed. Males fed a control diet were 10% heavier and displayed elevated interleukin (IL-18) (*p* = 0.01) in serum compared to all HMO-fortified groups at week 11. No differences in body composition were detected between groups. In females, HMOs did not affect body weight but 2′FL + 3′SL significantly increased cecum weight. All female HMO-fortified groups displayed significant reductions in intestinal permeability compared to controls (*p* = 0.02). All HMO-fortified diets altered gut microbiota composition and mRNA expression in the gastrointestinal tract, albeit differently according to sex. Supplementation with a fraction of the HMOs found in breast milk has a complex sex-dependent risk/benefit profile. Further long-term investigation of gut microbial profiles and supplementation with other HMOs during early development is warranted.

## 1. Introduction

Breast milk consumption during the first year of life is a crucial determinant of gastrointestinal tract and microbiota development [1]. Breast milk can enhance the intestinal barrier and reduce chronic disease risk later in life [2]; however, infant formula is now a commonly accepted alternative to breast milk. The infant formula market is anticipated to reach $98 billion by 2025 [3]. Based on trends, 39% of infants younger than six months of age were reported to be exclusively breastfed in developing countries between 1995 and 2010 [4] with substantial variability seen in developed countries with breastfeeding initiation rates of 38–97% [5].

Breast milk, when quantified as g/L, is composed of protein (10%), fats (30%), and carbohydrates (60%) [6]. A prominent amount of the carbohydrates present include important complex structures known as human milk oligosaccharides (HMOs). HMOs encompass a structurally diverse group of over 200 soluble carbohydrates [7], the composition of which varies throughout lactation and between mothers [8]. Oligosaccharides in human milk belong to three major classes, i.e., neutral fucosylated, neutral non-fucosylated, and acidic [9]. Despite their physical characterization, the functional significance of the various HMOs is still not fully understood. Although newborns lack the enzymatic capacity to break down HMOs [10], these oligosaccharides are utilized by commensal gut bacteria and act as prebiotics wherein they serve as a growth factor that promotes a healthy gut microbiota, thus improving host health [11]. HMOs were also shown to affect microbe interactions in the host by serving as decoy receptors to prevent pathogen binding to intestinal epithelial cells [12]. Likewise, HMOs affect matrix metalloproteinase genes, like MMP2 and MMP9, which modulate efficient barrier function and tight junction protein integrity, including occludin and zonula occluden (ZO)-1 [13]. Mucus is a major component of the physical intestinal barrier that contributes to intestinal protection and host defense [14]. HMOs are closely linked with intestinal mucins, whereby the mucus contributes to intestinal homeostasis by inhibiting bacterial adhesion to epithelial cells [15]. The mucus layer is constructed of MUC2 protein, of which the polysaccharide component can serve as an energy source for intestinal bacteria in the absence of fiber [16]. Further, HMOs directly stimulate MUC2 processing through protein disulfide isomerase (PDI) and maintain goblet cell populations [15]. MMP9, an extracellular proteinase, was shown to regulate MUC2 levels [13]. Similarly, G-protein-coupled receptors (GPCRs), like GPR41 and GPR43, have an important role in modulating intestinal inflammation [17,18].

Given that it is difficult to produce human breast milk on a large scale, it is becoming increasingly important to find a functional equivalent for infants fed infant formula with all the encompassing benefits of HMOs. Oligosaccharides isolated from bovine milk are substantially less abundant and structurally less complex compared to HMOs [19]. Therefore, no natural or synthetic source is able to reproduce the beneficial effects of human breast milk. Presently, some commercially available infant formulas are fortified with other prebiotics, most commonly galactooligosaccharides (GOS) and/or fructooligosaccharides (FOS). Importantly, GOS and FOS are neither fucosylated or sialylated, and they are structurally less complex than fucosylated or sialylated HMOs. Therefore, companies sought to synthesize and make commercially available milk oligosaccharides that are highly purified and structurally similar to those found in human milk. To mimic the presence of acidic and neutral HMOs found in breast milk, we chose to test the effects of supplementing a weanling diet in rats with two synthesized HMOs: the most abundant fucosylated HMO, 2′ fucosyllactose (2′FL) [20], as well as the most predominant sialyllactose that remains stable throughout lactation, 3′sialyllactose (3′SL) [21]. Our objective was to examine the effects of 2′FL and 3′SL alone or in combination on growth and body composition, gut microbiota, intestinal permeability, and expression of genes involved in gut health in young female and male rats.

## 2. Materials and Methods

### 2.1. Animal Model and Dietary Treatment

Twenty virgin female Sprague Dawley rats were obtained from Charles River Laboratories (Montreal, QC, Canada) and acclimatized to a temperature- and humidity-controlled facility with a 12-h light/dark cycle. After one week, females were bred in-house with male Sprague Dawley rats in wire-bottomed cages until a copulation plug was identified. Dams were then housed individually and fed a standard chow diet during gestation and lactation. The day after birth, pups were weighed, and litters were culled to 10 pups (five males; five females) to minimize differences in nutrition in litters of different sizes. Cross-fostering from dams with similar parturition dates was used to bring litters up to *n* = 10 pups when litters were <10 or the male–female ratio was imbalanced. At weaning, pups were randomized to one of four nutritionally complete experimental diets for eight weeks: (1) control (AIN-93G), (2) 2′ fucosyllactose-fortified AIN-93G (2′FL, 0.625% *wt*/*wt*), (3) 3′sialyllactose-fortified AIN-93G (3′SL, 0.625% *wt*/*wt*), and (4) 2′FL + 3′SL-fortified AIN-93G (0.625% *wt*/*wt* each). In total, *n* = 10 rats of each sex were allocated per diet group. An additional *n* = 10 rats per group of each sex were also included for the intestinal permeability test which had to be performed separately from the other tests. The dose of HMOs was chosen to provide a similar average dose of 2′FL that breastfed infants would obtain [20]. All diets were mixed in house using ingredients from Dyets, Inc. (Bethlehem, PA, USA) and HMOs from Glycom A/S (Hørsholm, Denmark). The composition of the experimental diets can be found in Table S1 (Supplementary Materials). Two animals per treatment (one for intestinal permeability test and one for all other outcomes) were co-housed per cage until week 10. Offspring were weighed weekly for eight weeks, and food intake was measured each week for three consecutive days at a time. This study was approved by the University of Calgary Animal Care Committee (AC14-0080).

### 2.2. Oral Glucose Tolerance Test

Five days prior to sacrifice, rats were fasted overnight for 12 h, and blood glucose was measured from a tail nick sample using a One Touch Ultra 2 glucose meter (Lifescan, Burnaby, Canada). Rats were gavaged with a 2 g/kg dose of glucose, and additional blood glucose measurements were made at 15, 30, 60, 90, and 120 min post gavage. At the 0-, 15-, 60-, and 120-min time points, additional blood was collected from the tail into chilled tubes containing diprotinin-A (0.034 mg/mL blood (MP Biomedicals, Irvine, CA, USA), Sigma protease inhibitor (1 mg/mL blood; Sigma Aldrich, Oakville, ON, Canada), and Roche Pefabloc (1 mg/mL of blood; Roche, Mississauga, ON, Canada). Samples were centrifuged, and the serum was stored at −80 °C until analysis for insulin.

### 2.3. Insulin Tolerance Test

Eight days prior to sacrifice, rats were fasted for 6 h and then administered a bolus of insulin (0.75 U/kg) via intraperitoneal injection. Glucose concentrations were measured immediately via tail nick at 0, 15, 30, 60, 90, and 120 min after the insulin injection using a One Touch Ultra 2 glucose meter.

### 2.4. Intestinal Permeability Test

Intestinal permeability was assessed using fluorescein isothiocyanate–dextran-4000 Da (FITC), average mol wt. 3000–5000 (Sigma-Aldrich, St. Louis, Missouri, USA). Given that the FITC is found in circulation throughout the body after it is administered, this test was performed in a separate set of male and female rats. Following a 6-h fast, rats received an oral gavage of FITC diluted with saline to 250 mg/mL (500 mg/kg bodyweight DX-4000-FITC). At 1 h post gavage, rats were anesthetized using isoflurane, and blood was collected via cardiac puncture of the left ventricle in a tube containing ethylenediaminetetraacetic acid (EDTA) (10 μL EDTA/mL of blood), stored on ice, and kept in the dark. Samples were centrifuged at 4 °C for 3 min (12,000× *g*); plasma was collected and then stored at −80 °C until analysis. Rats were subsequently overanesthetized and killed via aortic cut. At the time of analysis, plasma samples were diluted in equal volumes of PBS, and 50 uL of samples were loaded in duplicate onto a 96-well plate that contained standards made via serial dilution. FITC was measured using a fluorescence reader (FLX 800) at an emission wavelength of 535 nm and an excitation wavelength of 485 nm.

### 2.5. Final Body Composition, and Blood and Tissue Collection

One day prior to sacrifice, animals underwent a dual x-ray absorptiometry (DXA) scan (Hologic ODR 4500; Hologic Inc., Marlborough, MA, USA) under light anesthetic (isoflurane). Lean mass (g), fat mass (g), body fat %, bone mineral content (g), and bone mineral density (BMD) (g/cm^2^) were assessed using Hologic QDR software for small animals. Following 12 h of feed deprivation, rats were anesthetized with isoflurane and blood collected from the portal vein. From this sample, a Milliplex Rat Cytokine Array/Chemokine Array (Millipore, St. Charles, MO, USA) was used to measure serum TNF*α*, IL-1α, IL-1β, IL-5, IL-10, IL-18, and leptin (Eve Technologies, Calgary, AB, Canada). Rats were subsequently killed by overanesthetization and decapitation. The cecum, colon, and jejunum were excised, cleaned, weighed, snap-frozen, and stored at −80 °C.

### 2.6. Bacterial DNA Extraction and Microbiota Analysis

Fecal matter was collected at three, seven, and 11 weeks of age, snap-frozen, and stored at −80 °C. Microbial profiling was performed based on our previous work [22,23,24]. Briefly, bacterial DNA was extracted from ~60 mg of stool using the FastDNA spin kit for feces (MP Biomedicals, Lachine, QC, Canada). Half of the extracted sample was brought to a concentration of 4 ng/μL prior to storage at −20 °C for qPCR analysis, and the other half was used for 16S ribosomal RNA (rRNA) sequencing at the Center for Health Genomics and Informatics at the University of Calgary. Quantitative PCR (qPCR) was carried out as previously described [22,23,24] with primers that covered the major gut bacterial groups in rodents (Table S2, Supplementary Materials).

Bacterial community composition was assessed with Illumina’s 16S rRNA amplicon sequencing protocol of the V3 and V4 region on the MiSeq platform (Illumina, San Diego, CA, USA). Sequencing primers were removed using Cutadapt (version 1.16), and sequences were filtered for quality using the dada2 package (version 1.12) in R (version 3.5.3). A table of amplicon sequence variants (ASVs) was generated using dada and taxonomically classified using the Silva 132 database. Alpha diversity was calculated using Chao1, Shannon, and Simpson indices with the phyloseq package (version 1.24.2). Beta diversity was calculated using a principal coordinate analysis (PCoA) on a Bray–Curtis distance matrix containing ASVs present in at least 5% of the samples. Significance of alpha was set at 0.05.

### 2.7. Tissue Gene Expression Using Real-Time PCR

Total RNA was extracted from the proximal jejunum and proximal colon samples, and real-time PCR performed as previously described [25] with primers listed in Table S3 (Supplementary Materials). Gene expression was calculated using the 2^−∆Ct^ method. The jejunum was harvested 3 cm distal to the duodenojejunal flexure. The harvested proximal colon was composed of the ascending colon, terminating at the hepatic flexure.

### 2.8. Statistical Analysis

All data are presented as means ± standard error of the mean (SEM). Outcomes with a single time point (e.g., body fat, intestinal permeability, etc.) were assessed using a two-way ANOVA to determine the effects of diet and sex and their interaction. If there was a significant effect of sex, a one-way ANOVA with Tukey’s post hoc test was performed within males and females separately to determine differences across groups. Outcomes with multiple time points (e.g., body weight, oral glucose tolerance test (OGTT), etc.) were analyzed using repeated-measures ANOVA where time was used as the *within-subject* factor and diet and sex were the between-subject factors. If there was a significant sex effect, male and female data were analyzed separately. When a significant diet × time effect was identified, a one-way ANOVA with Tukey’s post hoc test was used to determine differences across groups. To assess correlations between a panel of inflammatory cytokines and messenger RNA (mRNA) expression of genes that maintain intestinal barrier function, a Pearson’s correlation analysis was conducted. Results were considered significant at *p* < 0.05. Statistics were performed with SPSS Statistics version 24.0 (IBM, Armonk, NY, USA).

## 3. Results

### 3.1. Bodyweight, Body Composition, Food Intake, and Serum Leptin

**Figure 1:**
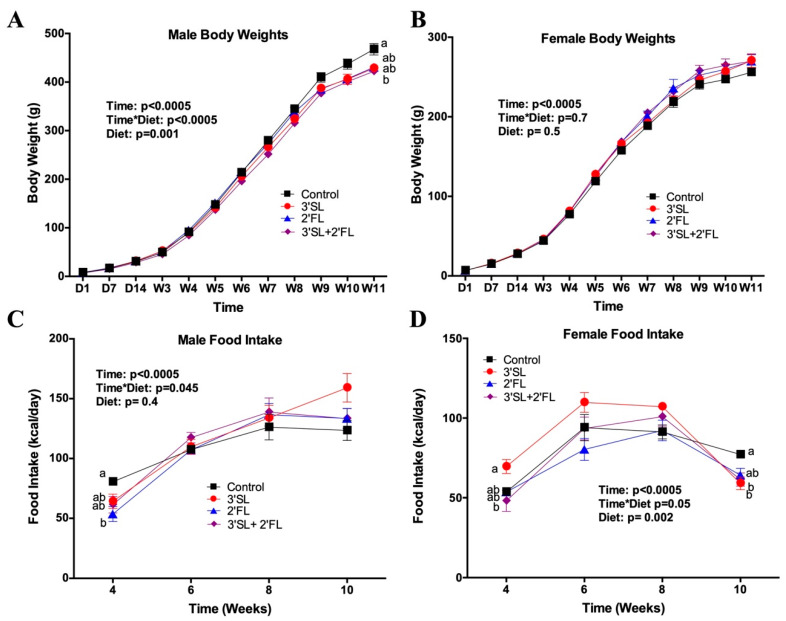
Body weight of (**A**) male and (**B**) female rats, as well as food intake for (**C**) male and (**D**) female rats fed the AIN-93 diet fortified with 3′sialyllactose (3′SL), 2′fucosyllactose (2′FL), both, or neither for eight weeks. Values are means ± standard error of the mean (SEM), *n* = 8–10. In the overall model, there was a significant sex effect for body weight (*p* = 0.001) and food intake (*p* = 0.001); therefore, subsequent analysis was performed in males and females separately. Within males and females, the superscripts a, b are used to depict differences between groups, where groups without a common superscript differ (*p* < 0.05). Control: AIN-93; 3′SL: AIN-93 + 3′sialyllactose; 2′FL: AIN-93 + 2′fucosyllactose; 3′SL + 2′FL: AIN-93 + 3′sialyllactose+ 2′fucosyllactose.

There was a significant effect of sex (*p* = 0.001) on body weight, with males weighing more than females at every age. Given the significant sex effect in the overall model, subsequent analysis was performed in males and females separately. Within the males, no differences in body weight were seen until week 11 when the control group was heavier than the 3′SL-fortified group (*p* = 0.03) (Figure 1A). There were no differences in body weight within females (Figure 1B).

There was a significant effect of sex (*p* = 0.0001) for food intake, with males consuming more food than females. Based on the significant sex differences in the overall model, male and female data were subsequently presented separately. Within the males and females, there was a significant main effect of time for food intake with intake increasing as the animals grew, as well as a significant interaction between time and diet in both males and females (Figure 1C,D). Males fed the control diet consumed significantly more kcal/day at four weeks of age compared to the 2′-FL-fortified group (*p* = 0.04). Females fed 3′SL consumed significantly more kcal/day at four weeks of age compared to the 3′SL + 2′FL group, and then eventually, by week 10, both 3′SL-fortified groups consumed significantly less energy than controls (*p* = 0.01).

Body composition was significantly affected by sex (*p* = 0.0001 for lean + bone mineral content (BMC), fat mass, % body fat, and BMC; *p* = 0.02 for bone mineral density). However, within males and females, there were no differences across diets in lean mass, fat mass, body fat %, and bone mineral density measured at 11 weeks of age (Table S4, Supplementary Materials). Serum leptin levels were significantly affected by sex (*p* = 0.001). Within males, serum leptin levels were significantly higher in rats fed control diet compared to 3′SL-fortified diet at 11 weeks of age (*p* = 0.03) (Figure S1A, Supplementary Materials). While not significant, male fat mass appeared to follow similar trends as serum leptin. To determine whether there was a relationship between fat mass and leptin levels, we conducted a correlation analysis stratified by sex and showed a significant positive association between male fat mass and serum leptin (*r* = 0.85, *p* = 0.0001) (Figure S2A, Supplementary Materials). No significant correlation was observed in females (Figure S2B, Supplementary Materials).

### 3.2. Intestinal Weight

Intestinal weight relative to body weight was significantly affected by sex (*p* = 0.0001). Within males, there were no differences across diets in cecum weight expressed per body weight at euthanasia at 11 weeks of age (Figure S3A, Supplementary Materials). Female cecum weight, however, was significantly higher in rats fed the 3′SL + 2′FL-fortified diet compared to controls (*p* = 0.002; Figure S3B, Supplementary Materials). There was no difference in male colon weight expressed per body weight; however, female colon weight was lower in the 3′SL + 2′FL-fortified group compared to the control group (*p* = 0.03) and 3′SL-fortified group (*p* = 0.02) (Figure S3C,D, respectively, Supplementary Materials).

### 3.3. Glucose and Insulin Tolerance Tests

**Figure 2:**
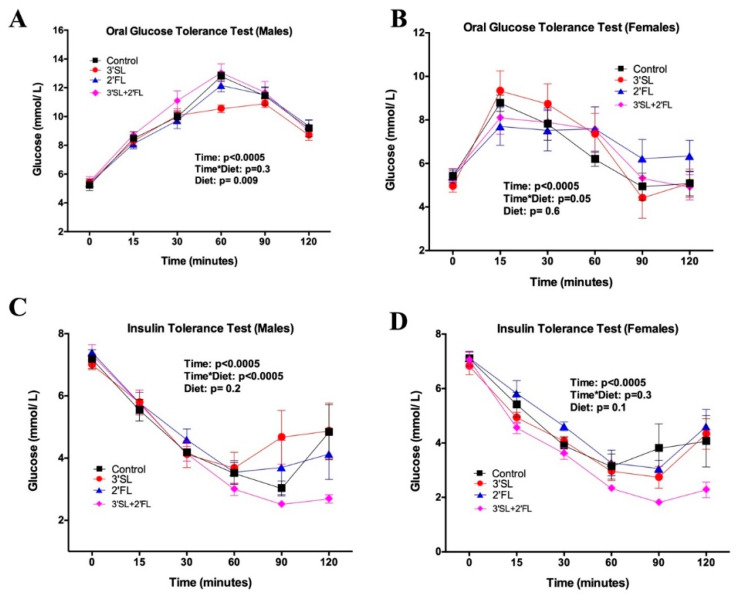
Oral glucose tolerance test (OGTT) in (**A**) male and (**B**) female rats; insulin tolerance test (ITT) in (**C**) male and (**D**) female rats fed the AIN-93 diet fortified with 3′SL, 2′FL, both, or neither for eight weeks. Values are means ± SEM, *n* = 8–10. In the overall model, there was a significant sex effect for OGTT (*p* = 0.001); therefore, subsequent analysis was performed in males and females separately. Control: AIN-93; 3′SL: AIN-93 + 3′sialyllactose; 2′FL: AIN-93 + 2′fucosyllactose; 3′SL + 2′FL: AIN-93 + 3′sialyllactose + 2′fucosyllactose. Significance was set at *p* < 0.05.

Blood glucose concentrations during the OGTT were significantly affected by sex (*p* = 0.0001). Given the significant sex effect in the overall model, subsequent analysis was performed in males and females separately. As expected, during an OGTT, there was a significant effect of time on glucose levels in males (Figure 2A) and females (Figure 2B) at the end of the eight-week feeding period. There was a significant independent effect of diet (*p* = 0.009) in males with the 3′SL-fortified group displaying lower glucose over time. No difference in AUC was observed in males (data not shown). In females, there was a trend toward an interaction between time and diet (*p* = 0.05).

During the ITTs, differences in blood glucose were limited to a significant effect of time in both males (Figure 2C) and females after log transformation (Figure 2D), as well as a significant interaction between time and diet (*p* < 0.0005) in males, wherein 3′SL + 2′FL showed the greatest insulin sensitivity but significant differences at individual time points could not be detected following Tukey’s post hoc testing.

### 3.4. Intestinal Permeability and Inflammatory Biomarkers

**Figure 3:**
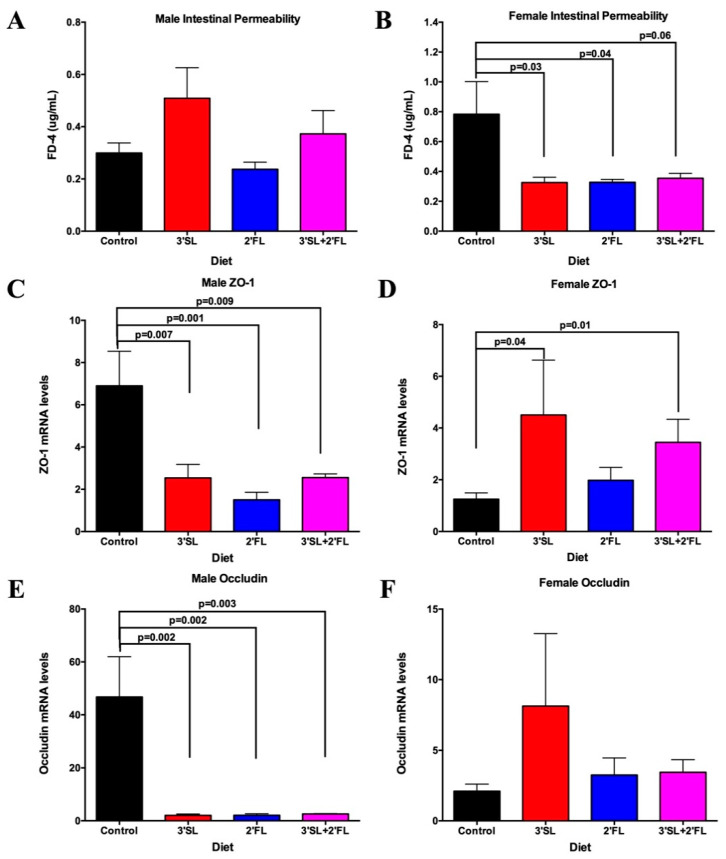
Intestinal permeability and jejunum mRNA levels. Plasma concentrations of fluorescein isothiocyanate (FITC) dextran 4000 (FD4) in (**A**) male and (**B**) female rats, as well as jejunum mRNA levels in (**C**) male zonula occluden-1 (ZO-1), (**D**) female ZO-1, (**E**) male occludin, and (**F**) female occludin in rats fed the AIN-93 diet fortified with 3′SL, 2′FL, both, or neither for eight weeks. Values are means ± SEM, *n* = 8–10. In the overall model, there was a significant sex effect for IPT (*p* = 0.03), ZO-1 (*p* = 0.001), and occludin (*p* = 0.0002); therefore, subsequent analysis was performed in males and females separately. Control: AIN-93; 3′SL: AIN-93 + 3′sialyllactose; 2′FL: AIN-93 + 2′fucosyllactose; 3′SL + 2′FL: AIN-93 + 3′sialyllactose + 2′fucosyllactose. Significance was set at *p* < 0.05.

In vivo intestinal permeability testing using FITC dextran 4000 (FD4) was significantly affected by sex (*p* = 0.03). There were no significant differences across diets in males (Figure 3A). In females, however, gut barrier permeability was reduced, as seen in lower plasma FITC concentrations, after log transformation in rats fed any of the HMO-fortified diets compared to controls (Figure 3B).

To assess whether this observed intestinal permeability was associated with changes in markers of systemic inflammation, we then examined a panel of serum inflammatory cytokines (Table S5, Supplementary Materials). There were significant sex effects for TNFα (*p* = 0.01), IL-5 (*p* = 0.001), and IL-18 (*p* = 0.0001) and a trend for IL-1β (*p* = 0.06) and IL-10 (*p* = 0.07) in the overall model; therefore, males and females were analyzed separately. In males, the 2′FL- and 2′FL + 3′SL-fortified groups had lower serum IL-18 concentrations compared to the control group, as well as trends showing reduced TNF*α* (*p* = 0.06) and IL-5 (*p* = 0.08) levels in all HMO-fortified groups compared to control male rats. No differences were observed in females; however, a trend (*p* = 0.07) was observed showing an increase in anti-inflammatory IL-10 concentrations in the 2′FL-fortified group.

### 3.5. Colon and Jejunum PCR

Based on changes in intestinal permeability, we then examined mRNA levels for select genes involved in gut-barrier function. There was a significant sex effect for certain genes (ZO-1, *p* = 0.001; occludin *p* = 0.0002); therefore, males and females were analyzed separately. In males, ZO-1 mRNA levels in the jejunum were significantly reduced in all HMO-fortified groups compared with controls (3′SL: *p* = 0.007; 2′FL: *p* = 0.001; 3′SL + 2′FL: *p* = 0.009) (Figure 3C). The opposite was observed in females; ZO-1 mRNA levels were higher in 3′SL (*p* = 0.04) and 3′SL + 2′FL (*p* = 0.01) groups, compared with controls (Figure 3D). Finally, male occludin gene expression in the jejunum was significantly reduced in all HMO-fortified groups compared with control (3′SL: *p* = 0.002; 2′FL: *p* = 0.002; 3′SL + 2FL: *p* = 0.003; Figure 3E). No difference was observed in females (Figure 3F).

**Figure 4:**
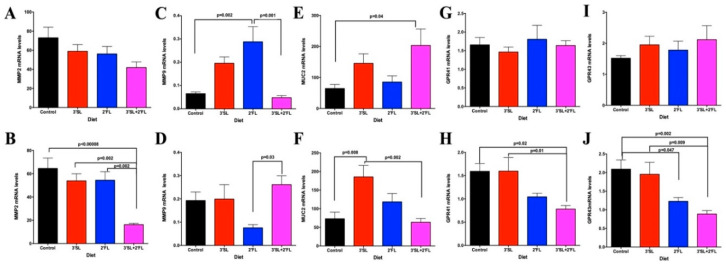
Proximal colon messenger RNA (mRNA) levels of (**A**) matrix metalloproteinase 2 (MMP2), (**C**) MMP9, (**E**) mucin 2 (MUC2) (**G**) G-protein-coupled receptor 41 (GPR41), and (**I**) GPR43 in male and (**B**) MMP2, (**D**) MMP9, (**F**) MUC2, (**H**) GPR41, and (**J**) GPR43 in female rats fed the AIN-93 diet fortified with 3′SL, 2′FL, both, or neither for eight weeks. Values are means ± SEM, *n* = 8–10. Control: AIN-93; 3′SL: AIN-93 + 3′sialyllactose; 2′FL: AIN-93 + 2′fucosyllactose; 3′SL + 2′FL: AIN-93 + 3′sialyllactose + 2′fucosyllactose. Significance was set at *p* < 0.05.

In the proximal colon, no differences were observed in male MMP2 mRNA levels (Figure 4A); however, females exhibited significantly reduced MMP2 mRNA levels in the 3′SL + 2′FL group compared to all other groups (Figure 4B).

MMP9 mRNA levels in males were higher in the 2′FL-fortified group compared to the control and 3′SL + 2′FL-fortified groups (*p* = 0.002 and 0.001, respectively; Figure 4C). In females, MMP9 mRNA levels were higher in the 3′SL + 2′FL-fortified group compared to the 2′FL group (*p* = 0.03; Figure 4D). MUC2 gene expression in males was significantly higher in 3′SL + 2′FL-fortified group compared to control (*p* = 0.04) (Figure 4E). The opposite was true for females, where lower MUC2 mRNA levels were seen in 3′SL + 2′FL group compared to the 3′SL-fortified group (*p* = 0.002), while 3′SL-fortified group MUC2 mRNA levels were higher compared to control (*p* = 0.008; Figure 4F).

No differences were observed in male GPR41 and GPR43 mRNA levels in the proximal colon (Figure 4G,I respectively); however, female GPR41 (Figure 4H) and GPR43 (Figure 4J) mRNA levels were decreased in 3′SL + 2′FL-fortified groups compared with controls and the 3′SL group (GPR41: *p* = 0.02 and 0.01, respectively; GPR43: *p* = 0.002 and 0.009, respectively). The 2′FL- fortified group also showed a marked reduction compared with controls in GPR43 mRNA levels (*p* = 0.047).

To assess whether there was a relationship between inflammatory cytokines and genes associated with barrier function, we conducted a correlation analysis. Males exhibited a significant positive correlation between circulating IL-18 and mRNA levels of tight junction proteins ZO-1 and occludin (Table S6, Supplementary Materials). Females displayed a significant positive correlation between IL-18 and MUC2 mRNA levels (Table S7, Supplementary Materials). Knowing that IL-18 was shown to disrupt tight junctions in gastrointestinal epithelial monolayers [26], we further investigated the seemingly contradictory positive correlation between IL-18 and ZO-1 and occludin mRNA levels in males. Given that male control rats had approximately double the concentration of serum IL-18 as the 2′FL group, we stratified according to group and found a significant negative correlation between IL-18 and ZO-1 mRNA levels in the 2′FL group (*r* = −0.838; *p* < 0.001), which is consistent with previous findings [26].

### 3.6. Gut Microbial Profiling: qPCR and 16S rRNA Sequencing

Based on differences in gut epithelial gene expression in HMO-supplemented rats, we proceeded to examine the gut microbial profile of fecal samples right after weaning (start of the diet intervention), at seven weeks of age (week 4 of the intervention), and at 11 weeks of age (week 8 of the intervention). Due to a significant effect of sex for certain bacterial groups (e.g., *Lactobacillus* spp. *p* = 0.002), males and females were analyzed separately.

Using qPCR, at seven weeks of age, males showed significantly higher abundance of total bacteria in the 3′SL-fortified group compared with controls (*p* = 0.02) (Table 1). The relative abundances of *Clostridium* cluster I and *Clostridium* cluster XI were significantly reduced in all HMO-fortified groups compared to controls (*p* = 0.0004 and 0.002, respectively). *Clostridium* cluster IV was reduced in the 2′FL-fortified group compared to control (0.04). *Bifidobacterium* spp. were significantly higher in the 2′FL group compared to the 3′SL-fortified group (*p* = 0.03). *Akkermansia muciniphila*, after log transformation, showed a significant reduction in the 3′SL + 2′FL group compared with control (*p* = 0.01).

At 11 weeks of age, qPCR analysis showed that males had an increase in the relative abundance of *Roseburia* spp. in the 3′SL-fortified group compared to all other groups (*p* = 0.01; Table S8, Supplementary Materials), as well as a reduction in *Enterobacteriaceae* in the 2′FL-fortified group compared to control (*p* = 0.02; Table S8, Supplementary Materials).

In female rats, at seven weeks of age, qPCR analysis showed that *Akkermansia muciniphila* spp. were significantly reduced in all HMO-fortified groups compared to control (*p* = 0.04; Table 2).

At 11 weeks of age, qPCR analysis showed numerous differences between groups in female rats (Table S9, Supplementary Materials). The total level of bacteria was higher in the 2′FL compared with the 3′SL + 2′FL-fortified group (*p* = 0.018), and it showed a trend toward being higher than all other groups. *Clostridium* cluster I abundance was increased in the 2′FL-fortified group compared to groups fortified with 3′SL, alone or in combination (*p* = 0.004 and *p* = 0.007). *Methanobrevibacter* spp. were significantly reduced in groups fortified with 2′FL, alone or in combination, compared to the 3′SL group. (*p* = 0.03 and *p* = 0.03, respectively). *Akkermansia muciniphila*, after log transformation, was significantly reduced in the 3′SL + 2′FL-fortified diet group compared to control (*p* = 0.004). 

Based on 16S rRNA sequencing, HMO-fortified diets showed no effect on alpha diversity according to Shannon or Chao1 indices in both males and females (data not shown). However, the Simpson index in males showed higher alpha diversity in the 3′SL + 2′FL-fortified group at 11 but not three or seven weeks (*p* = 0.02; Figure S4, Supplementary Materials). No difference was seen in females at three, seven, or 11 weeks of age (Figure S4, Supplementary Materials). No difference in beta diversity was seen between groups (Figure S5, Supplementary Materials).

**Figure 5:**
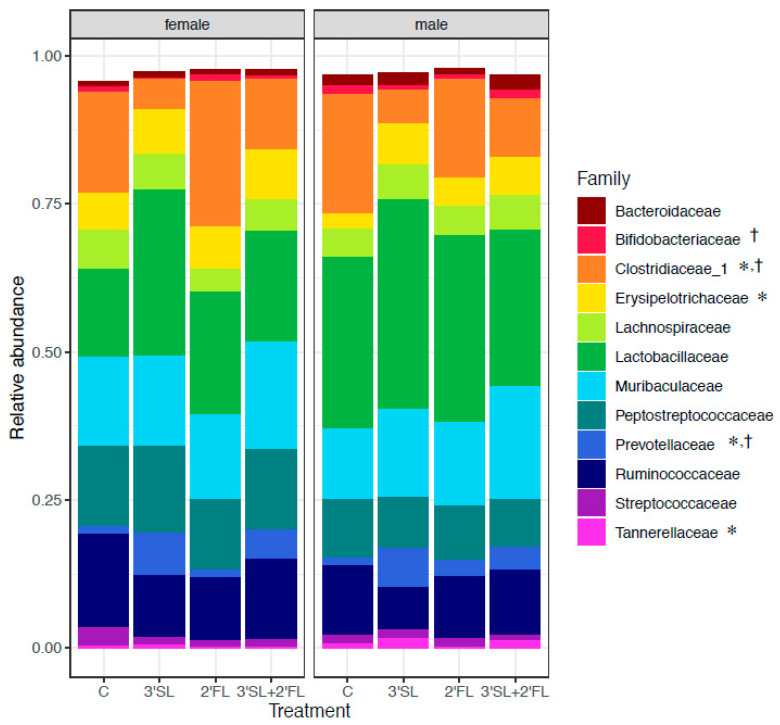
Relative abundance plots of bacterial taxa using 16S rRNA sequencing in male and female rat feces at 11 weeks of age. Taxa were identified to the taxonomic level of family using the Silva reference database. Control: AIN-93; 3′SL: AIN-93 + 3′sialyllactose; 2′FL: AIN-93 + 2′fucosyllactose; 3′SL + 2′FL: AIN-93 + 3′sialyllactose + 2′fucosyllactose. * *p* < 0.05 (males); † *p* < 0.05 (females).

Complementary to qPCR data, 16S rRNA sequencing data in males showed that *Clostridiaceae_1* relative abundance was reduced in the 3′SL and 3′SL + 2′FL groups compared with controls in both males and females (Figure 5 and Figures S6 and S7, Supplementary Materials). *Prevotellaceae* relative abundance was significantly lower in the 2′FL group compared to the 3′SL and 3′SL + 2′FL groups in both males and females (Figure 5 and Figures S6 and S7, Supplementary Materials). Similarly, *Erysipelotrichaceae and Tannerellaceae* relative abundance in males was higher in groups fortified with 3′SL, alone or in combination. In females, *Bifidobacteriaceae* relative abundance was highest in the 2′FL group compared to the 3′SL group.

## 4. Discussion

Human milk is a highly evolved, structurally complex, complete biomaterial that nourishes developing infants, while simultaneously acting as a growth factor, prebiotic, modulator of gut microbiota/gut barrier function, and immune regulatory factor [27]. Breastfeeding for less than 4–6 months or not at all is associated with greater incidence of immune-mediated diseases, infectious diseases, overweight, obesity, and other metabolic ailments in adulthood [28]. This is in part due to the absence of important HMOs like 3′SL and 2′FL. A complete HMO profile provides a biological advantage within the gastrointestinal tract and throughout the body [27]. To our knowledge, this is the first study looking at the fortification of a weanling diet with the HMOs, 3′SL and 2′FL, alone or in combination, and how they may impact gut microbial composition, intestinal permeability, inflammatory cytokines, and intestinal gene expression in males and females. HMO supplementation in females improved intestinal permeability, mRNA expression of important genes involved in maintaining gut barrier function, and gut microbial composition. Males supplemented with HMOs displayed reductions in weight gain at the end of an eight-week intervention, improved pro-inflammatory cytokine profiles, and an increased abundance in beneficial gut microbes.

HMOs cannot be digested by the human infant; they are primarily considered prebiotics, denoting their indigestible nature and selective utilization by beneficial gut microbes. Using a chemically defined medium, facilitating vigorous growth of gut-related microbes, researchers found that some strains of *Bifidobacterium* and *Bacteroides* are able to utilize HMOs with high efficiency [29]. A dysbiotic gut at an early age may be predictive of disease later in life. Breast-fed infants harbor a distinct gut microbiota, dominated by bifidobacteria [30]. Our study found that neutral 2′FL compared to 3′SL and control diets enhanced this bifidogenic effect at seven weeks of age in males and 11 weeks of age in females. This observation might be indicative of the genetic capability of select bacteria co-evolving with HMOs to enable their utilization [27]. Bifidobacteria strains, for example, utilize varied oligosaccharides as growth substrates [31]; 2′FL, as noted in our study in both males and females, may be one of them. Other researchers found that *Bifidobacterium infantis* utilize HMOs lacto-*N*-tetraose (LNT) and lacto-*N*-neotetrose (LNnT) [31]. Inefficient metabolism of these HMOs will result in a deleterious shift in *B. longum* subsp. *infantis* physiology, thereby impacting offspring health [31]. Another example is disialyllactose-*N*-tetraose (DSLNT), which is the most effective HMO to reduce necrotizing enterocolitis-like symptoms in a neonatal rat model [32]. *B. longum* ssp. *infantis* ATCC 15,697 and B. *infantis* M-63 are the only two microbes known to be able to ferment 3′SL, 6′SL, 2′FL, and 3′FL, with the latter able to degrade about 90% of 2′FL [33]. Furthermore, out of all bifidobacteria strains, only *B. infantis* species and *B. breve* ATCC 15,700 are able to ferment LNnT, while *L. acidophilus* NCFM, among lactobacilli, utilize LNnT most efficiently [33]. It is important to note that, while we saw differences in bifidobacteria abundance in male and female Sprague Dawley rats, we are not aware of research in which HMO utilization by bifidobacteria from the rodent gut was shown directly.

A growing body of evidence suggests that, in the infant gut, there exist a multitude of HMO-adapted microbes like bifidobacteria and *Bacteroides* [29,33]. Therefore, it becomes increasingly vital to determine which of the over 200 identified HMOs are responsible for beneficial effects metabolically, immunologically, cognitively, or otherwise. This characterization would be vital in selecting individual HMOs for supplementation purposes. In vitro incubations of multiple strains of bifidobacteria using lacto-*N*-biose (LNB) or sialyllactose indicate that only a select few species are able to proliferate using isolated HMOs as a carbon source [34,35]. *B. infantis* grows on HMOs as a sole sugar source, whereas *L. gasseri* does not [36].

*Akkermansia muciniphila* is known for its mucin-utilizing characteristics [37]. Using a comprehensive array of techniques to analyze and differentiate between all the bacteria in the intestinal tract, *A. muciniphila* was uniquely found to reach 100 times its original abundance following prebiotic ingestion, which corresponds with an improved metabolic profile [38]. It is important to note that this discovery was only observed in genetic or diet-induced obese mice [38]. Our findings showed a 10- to 30-fold reduction in *A. muciniphila* in females fed 3′SL + 2′FL-fortified formula at seven and 11 weeks of age, respectively, despite improvements in tight junction protein expression in the jejunum, as well as observed reductions in intestinal permeability in the colon as demonstrated by lower FITC in all HMO-supplemented groups in females. This is likely because, unlike previously published findings, showing a 100-fold increase, our rodents were neither genetic nor diet-induced obese animals. In fact, we found no changes in body weight, fat or lean mass, or insulin resistance between groups; therefore, *A. muciniphila* relative abundance perhaps only increases in metabolically overweight or obese models. It is possible that *A. muciniphila* is only needed to reverse HFD-induced metabolic disorders and improve intestinal barrier function. Supporting these findings, we also found a concurrent increase in the relative abundance of the Verrucomicrobia phylum (data not shown), of which *A. muciniphila* is a member, using 16S rRNA sequencing technology.

Clostridia and enterococci are characterized as non-HMO consumers using a chemically defined medium, ZMB1 [29], explaining why our study found significantly reduced abundance of Enterobacteriaceae in the 2′FL-fortifed group, alone or in combination with 3′SL, and a trend of reduced presence of *C. difficile* in the 2′FL and 3′SL + 2′FL-fortified male groups at 11 weeks of age.

Increased intestinal permeability is postulated to be resultant of reduced expression of tight junction proteins [39]. Tight junctions are made up of claudins, occludins, and zonula occludens (ZO)-1, 2, 3, which regulate the paracellular permeability of endothelial and epithelial cells, while also operating as a barrier against bacterial invasion [40]. Importantly, tight junction proteins show sex-dependent expression and modulation. One study examined the expression of estrogen receptor (ER-α/β) and ZO-1 in male and female gut tissues, as well as concurrent inflammatory activation in vitro [41]. They found that ZO-1 expression was significantly lower in female compared to male tissue, and estrogen treatment decreased ZO-1 mRNA and protein expression, signifying that sex hormones may regulate tight junction proteins in the gut [41]. This differential expression could explain why we saw distinct sex differences in intestinal permeability in males and females. The increased expression of ZO-1 in 3′SL HMO-fortified groups, alone or in combination with 2′FL, uniquely reduced intestinal inflammation in females. ZO-1 maintains a selectively permeable epithelial barrier and impedes the translocation of bacterial populations into circulation from the intestinal lumen. HMO-fortified diets, in females at least, appear to maintain these tight junction proteins and reduce intestinal permeability approximately three-fold compared to control. A 2009 study using the lactulose/mannitol test found similar changes, where breast-fed infants showed a 2.8-fold reduction in intestinal permeability compared to exclusively formula fed infants [2]. Two early studies in term infants, also examining different feeding types, showed reduced intestinal permeability in breast-fed compared to formula-fed infants but at different periods postnatally [42,43]. Rats fed a high-fat diet for a prolonged period of time showed increased intestinal permeability, as well as a dysbiotic microbiota which was shown to be prevented with the addition of bovine milk oligosaccharides (BMO) and prebiotic inulin [44]. Inulin, like milk oligosaccharides, is a prebiotic and a soluble fiber. Supplementation with soluble fibers is known to ameliorate gut dysbiosis and reduce low-grade inflammation, typically linked, at least in part, with decreased intestinal permeability [45,46].

HMOs are associated with anti-inflammatory effects by affecting cytokine production, the initial change from an intrauterine Th2 prevailing-response to a Th1/Th2 balanced one [47], and the inhibition of leukocyte rolling and adhesion to endothelial cells under variable conditions [48]. Among these cytokines, IL-18 is traditionally considered a pro-inflammatory cytokine produced by a myriad of structures, including lactating mammary glands and intestinal epithelial cells [49]. In human milk, preterm delivery or complications during pregnancy are associated with higher levels of IL-18 in breast milk [49]. Our findings show reductions in serum IL-18 and a trend toward a decrease in TNF*α* and IL-5 in male groups fortified with 2′FL HMOs, alone or in combination with 3′SL. Validating these findings, a randomized controlled trial found that healthy infants born at term, fed formula fortified with 2′FL, had reductions in pro-inflammatory cytokines, as measured ex vivo in plasma and mirroring those of breast-fed infants [50]. Furthermore, comparable in vivo differences in cytokine levels were found in allergy-prone infants in the first year of life between breast-fed versus formula-fed infants [51]. Pu et al. [52] recently suggested a dual function of IL-18, primarily in a colitis model, whereby IL-18 may have pro- or anti-inflammatory functions. They found that IL-18 treatment at an earlier stage of colitis changed colon length, reduced inflammatory infiltration, and increased Muc2 expression [52]. This potentially explains why we saw a positive correlation between IL-18 and MUC2 expression in our young, healthy females, as well as a reduction in intestinal permeability across all HMO-fortified groups.

To further explore a link with inflammation, we examined GPR41 and GPR43 mRNA expression. Short-chain fatty acids (SCFA) bind to these receptors to modulate intestinal inflammation, by reducing the secretion of proinflammatory cytokines and chemokines [53]; however, GPR41 and GPR43 mRNA expression in males and females in our study did not mirror our serum inflammatory cytokine data. As such, it would be important in future studies to examine intestinal histology (infiltration, crypt alterations, erosion, etc.) and expression of inflammatory genes in a sex-dependent manner to determine if the HMOs indeed affect inflammation at the intestinal level. Estrogen has an anti-inflammatory effect due to inhibition of nuclear factor kappa B (NF-κB) activation [54], which may explain the important sex differences in serum inflammatory cytokines, where females showed a trend toward an increase in anti-inflammatory cytokine IL-10 in the 2′FL-HMO-fortified group.

Our study demonstrated that HMO supplementation of 3′SL or 2′FL alone or in combination elicits distinct sex differences which may be positive or negative. Previous reports combined data from both sexes, failing to distinguish important sex differences in metabolic outcomes. There is also growing evidence showing that sex is an important factor to consider when examining interactions between gut microbiota and environmental factors such as diet, and not stratifying by sex can obscure important sex-by-diet interactions [55]. To our knowledge, we are the first group to demonstrate important sex differences after 3′SL and 2′FL HMO supplementation. We established that females experienced improvements in gut morphology and barrier function, as well as overall improvements in gut microbial composition at the family taxonomic level. In males, however, 3′SL and 2′FL HMO supplementation resulted in patterns of mRNA levels in the jejunum and colon, including ZO-1, occludin, and MMP9 that are commonly associated with compromised gut permeability, although MUC2 mRNA levels showed upregulation with HMOs. In the future, it would be important to examine protein levels of these genes and other indices of gut barrier function to fully understand the implications of the changes in gene expression identified. Males also displayed slightly lower weight gain and inflammatory biomarkers during the final week of the intervention compared to control, as well as an increased abundance of beneficial gut microbes at varying taxonomic levels. Nevertheless, our study is not without limitations. We found apparent conflicting findings resultant of the supplementation of 3′SL and 2′FL alone or in combination, which may be owing to changes in HMO metabolism within the gut during postnatal development. Based on fecal oligosaccharide profiles, HMO metabolism is postulated to progress through three stages in human infants [56]: (1) from birth to two months, prevalence of neutral or acidic oligosaccharide metabolism (a seven-day-old rat is approximately equivalent to a newborn human infant in terms of central nervous system and reproductive development); (2) at 2–4 months, reduction of HMOs in infant fecal matter and an increase in HMO metabolites (approximately equivalent to rats in the week leading up to weaning); (3) four months onward, when complementary solid foods begin introduction, a substantial reduction of HMOs, as well as their metabolites, and an increase in oligosaccharides, typically complimenting the introduction of solid foods (approximately equivalent to 21 days and onward in rats). Our study introduced HMOs at approximately stage 2 of HMO metabolism and continued these interventions well past stage 3. In stage 3, we maintained a single standard diet, AIN-93 and HMO-fortification; therefore, it is likely that HMOs and their metabolites would have remained consistent in fecal matter throughout the intervention, although perhaps eliciting less than beneficial effects [57]. Alternatively, findings from a randomized controlled trial of healthy term infants given the HMOs 2′FL and LNnT shifted the gut microbiota toward that of breast-fed infants. We did not supplement our rodents with LNnT HMO which may be why we did not observe similar effects. Finally, we acknowledge that we only supplemented a small fraction of these HMOs, which are unlikely to provide the exact benefits conferred from the evolutionary forces, perfecting the process of exclusive breastfeeding, ensuring the greatest health benefit for the infant [29]. Future studies should investigate whether the addition of *Lactobacillus*, *Bifidobacterium*, and *Bacteroides* combined with more HMOs, including 3′SL, 2′FL, and LNnP, in the formula will elicit similar immunoregulatory and symbiotic gut microbial proliferation as breast-fed infants, stratified by sex.

## Figures and Tables

**Table 1:** Relative abundance of fecal microbiota (qPCR) in male rats at seven weeks of age fed the AIN-93 diet fortified with 3′SL, 2′FL, both, or neither.

Treatment	Control	3′SL	2′FL	3′SL + 2′FL	*p*-Value
	Relative Abundance (%)				
***Bacteroides/Prevotella* spp.**	1.9 ± 0.3	1.4 ± 0.2	1.7 ± 0.3	1.3 ± 0.2	0.66
***Bifidobacterium* spp.**	0.9 ± 0.2 ^ab^	0.8 ± 0.2 ^a^	3.1 ± 1.0 ^b^	2.3 ± 0.4 ^ab^	0.03
***Enterobacteriaceae***	0.2 ± 0.04	0.2 ± 0.1	0.1 ± 0.02	0.1 ± 0.02	0.31
***Lactobacillus* spp.**	47.1 ± 6.6	57.4 ± 9.2	43.7 ± 9.6	38.2 ± 9.5	0.48
***Clostridium perfringens* (cluster I)**	1.5 ± 0.2 ^a^	0.7 ± 0.2 ^b^	0.8 ± 0.2 ^b^	0.6 ± 0.1 ^b^	0.0004
***Clostridium leptum* (cluster IV)**	9.1 ± 1.5 ^a^	5.1 ± 1.0 ^ab^	4.7 ± 0.9 ^b^	7.1 ± 1.2 ^ab^	0.04
***Clostridium difficile* (cluster XI)**	0.4 ± 0.1 ^a^	0.1 ± 0.03 ^b^	0.1 ± 0.02 ^b^	0.1 ± 0.01 ^b^	0.002
***Clostridium coccoides* (cluster XIV)**	16.3 ± 2.3	10.6 ± 1.4	10.4 ± 2.1	13.5 ± 1.9	0.12
***Roseburia* spp.**	0.003 ± 0.002	0.004 ± 0.002	0.0006 ± 0.0001	0.003 ± 0.001	0.36
***Methanobrevibacter* spp.**	0.005 ± 0.003 ^a^	0.003 ± 0.0003 ^b^	0.003 ± 0.0001 ^b^	0.004 ± 0.001 ^ab^	0.001
***Akkermansia muciniphila***	0.07 ± 0.03 ^a^	0.04 ± 0.01 ^ab^	0.02 ± 0.007 ^ab^	0.003 ± 0.001 ^b^	0.01
***Faecalibacterium prausnitzii***	0.09 ± 0.03	0.03 ± 0.008	0.04 ± 0.008	0.05 ± 0.005	0.20
***Collinsella aerofaciens***	0.005 ± 0.0004 ^a^	0.003 ± 0.0003 ^b^	0.003 ± 0.001 ^b^	0.004 ± 0.001 ^ab^	0.002
**Total bacteria** (16S ribosomal RNA (rRNA) gene copies)	30,736,150 ± 2,698,774 ^a^	53,509,340 ± 6,514,451 ^b^	49,386,585 ± 4698662 ^ab^	38,298,292 ± 5,529,141 ^ab^	0.02

Values are means ± SEM, *n* = 8–10. *Bacteriodes/Prevotella* spp., *Enterobacteriaceae*, *A. muciniphila*, and *F. prausnitzii* were log-transformed for analysis. Total bacteria are represented as 16S rRNA gene copies/20 ng genomic DNA. All other taxa are presented as relative abundance (%) of bacterial taxa per total bacteria (16S rRNA gene copies/total 16S rRNA gene copies). Control: AIN-93; 3′SL: AIN-93 + 3′sialyllactose; 2′FL: AIN-93 + 2′ fucosyllactose; 3′SL + 2′FL: AIN-93 + 3′sialyllactose + 2′ fucosyllactose. The superscripts ^a,b^ are used to depict differences between groups, where groups without a common superscript differ (*p* < 0.05).

**Table 2:** Relative abundance of fecal microbiota (qPCR) in female rats at seven weeks of age fed the AIN-93 diet fortified with 3′SL, 2′FL, both, or neither.

Treatment	Control	3′SL	2′FL	3′SL + 2′FL	*p*-Value
	Relative Abundance (%)				
***Bacteroides/Prevotella* spp.**	1.2 ± 0.1	1.9 ± 0.3	1.8 ± 0.3	1.4 ± 0.04	0.11
***Bifidobacterium* spp.**	0.9 ± 0.4	1.2 ± 0.4	1.7 ± 0.5	1.3 ± 0.3	0.34
***Enterobacteriaceae***	0.1 ± 0.02	0.2 ± 0.04	0.1 ± 0.02	0.05 ± 0.009	0.06
***Lactobacillus* spp.**	41.7 ± 5.3	42.0 ± 8.1	45.2 ± 5.6	45.7 ± 10.1	0.97
***Clostridium perfringens* (cluster I)**	1.0 ± 0.1	1.3 ± 0.2	1.3 ± 0.2	1.2 ± 0.06	0.60
***Clostridium leptum* (cluster IV)**	6.5 ± 1.1	7.1 ± 1.3	10.7 ± 1.7	6.7 ± 1.2	0.10
***Clostridium difficile* (cluster XI)**	0.1 ± 0.03	0.1 ± 0.03	0.1 ± 0.01	0.08 ± 0.02	0.35
***Clostridium coccoides* (cluster XIV)**	10.9 ± 1.6	11.6 ± 1.5	12.2 ± 1.1	17.6 ± 2.8	0.06
***Roseburia* spp.**	0.001 ± 0.0004	0.0006 ± 0.0001	0.0009 ± 0.0002	0.003 ± 0.001	0.20
***Methanobrevibacter* spp.**	0.004 ± 0.001	0.005 ± 0.001	0.004 ± 0.001	0.006 ± 0.001	0.94
***Akkermansia muciniphila***	0.1 ± 0.07 ^a^	0.02 ± 0.006 ^b^	0.03 ± 0.01 ^b^	0.009 ± 0.004 ^b^	0.04
***Faecalibacterium prausnitzii***	0.07 ± 0.02	0.05 ± 0.01	0.04 ± 0.006	0.05 ± 0.009	0.61
***Collinsella aerofaciens***	0.003 ± 0.001	0.004 ± 0.001	0.004 ± 0.0004	0.004 ± 0.001	0.36
**Total bacteria** (16S rRNA gene copies)	47,474,463 ± 3,993,791	47,238,918 ± 6,656,759	47,704,183 ± 2,956,712	33,004,782 ± 4,106,583	0.10

Values are means ± SEM, *n* = 8–10. *Bacteroides/Prevotella* spp., *Bifidobacterium* spp., *Enterbacteriaceae*, *Roseburia* spp., *Methanobrevibacter* spp., and *F. prausnitzii* were log transformed for analysis. Total bacteria are represented as 16S rRNA gene copies/20 ng genomic DNA. All other taxa are presented as relative abundance (%) of bacterial taxa per total bacteria (16S rRNA gene copies/total 16S rRNA gene copies). Control: AIN-93; 3′SL: AIN-93 + 3′sialyllactose; 2′FL: AIN-93 + 2′ fucosyllactose; 3′SL + 2′FL: AIN-93 + 3′sialyllactose + 2′ fucosyllactose. The superscripts ^a,b^ are used to depict differences between groups, where groups without a common superscript differ (*p* < 0.05).

## Supplementary Materials

The following are available online at https://www.mdpi.com/2072-6643/12/5/1532/s1: Table S1. Experimental diet composition from weeks 3–9 and 10–11; Table S2. Gut microbial group specific primers for qPCR; Table S3. Primer sequences for RT-PCR; Table S4. Male and female body composition; Table S5. Male and female serum inflammatory cytokines at 11 weeks of age; Table S6. Correlation analysis in males for a panel of inflammatory cytokines and mRNA levels that maintain intestinal barrier function; Table S7. Correlation analysis in females for a panel of inflammatory cytokines and mRNA levels that maintain intestinal barrier function; Table S8. Relative abundance of fecal microbiota (qPCR) in male rats at 11 weeks; Table S9. Relative abundance of fecal microbiota (qPCR) in female rats at 11 weeks of age; Figure S1. Serum fasting leptin levels; Figure S2. Correlation analysis between fat mass and leptin; Figure S3. Cecum and colon weight; Figure S4. Male and female alpha diversity using the Simpson index; Figure S5. Male and female beta diversity at 11 weeks of age; Figure S6. Heatmap of top gut bacterial phyla, families, and genera between treatments in males at 11 weeks of age; Figure S7. Heatmap of top gut bacterial phyla, families, and genera between treatments in females at 11 weeks of age.
