# Supplementary material for: Human Milk Oligosaccharide Supplementation Affects Intestinal Barrier Function and Microbial Composition in the Gastrointestinal Tract of Young Sprague Dawley Rats
Source: Nutrients. 2020 May 25;12(5):1532. doi: 10.3390/nu12051532 (PMC7284880; doi:10.3390/nu12051532)
Supplement: Supplementary file 1 [file nutrients-12-01532-s001.pdf]

Supplementary Material

Table S1: Experimental diet composition from weeks 3-9 and 10-11

	**Control**	**3'SL**	**2'FL**	**2'SL+2FL**	**Control**	**3'SL**	**2'FL**	**2'SL+2FL**
**g/kg**	**Weeks 3-9**				**Weeks 10-11**			
**Cornstarch**	397.5	397.5	397.5	397.5	465.7	465.7	465.7	465.7
**Casein**	200	200	200	200	140	140	140	140
**Dyetrose**	132	132	132	132	155	155	155	155
**Sucrose**	100	100	100	100	100	100	100	100
**Soybean Oil**	70	70	70	70	40	40	40	40
**Alphacel**	50	50	50	50	50	50	50	50
**AIN-93M Mineral Mix**	35	35	35	35	35	35	35	35
**AIN-93 VX Vitamin Mix**	10	10	10	10	10	10	10	10
**L-cystine**	3	3	3	3	1.8	1.8	1.8	1.8
**Choline-Bitartrate**	2.5	2.5	2.5	2.5	2.5	2.5	2.5	2.5
**3'Sialyllactose**	-	6.25	-	6.25	-	6.25	-	6.25
**2'Fucosyllactose**	-	-	6.25	6.25	-	-	6.25	6.25
**Energy density (kJ/g)**	15.73	15.69	15.69	15.64	15.07	15.04	15.04	14.99
**Carbohydrate (% of kcal)**	63.9	64.0	64.0	64.1	75.9	76.0	76.0	76.1
**Protein (% of kcal)**	19.4	19.3	19.3	19.2	14.1	14.1	14.1	14.0
**Fat (% of kcal)**	16.8	16.7	16.7	16.6	10.0	10.0	10.0	9.9

All diets were mixed in house using ingredients from Dyets Inc. (Bethlehem, PA, USA) and HMOs from Glycom A/S (Hørsholm, Denmark). Diet composition from weeks 3-9 is based on the AIN-93G formulation to support growth while the diet from weeks 10-11 is based on AIN-93M for adult maintenance. The purity of 2'FL is 96.1% (w/w%) and of 3'SL is 97.5% (w/w%).

Table S2: Gut microbial group specific primers for qPCR

Microbial Group	Primers (5'→3')	
**Firmicutes:**		
Clostridium difficile (cluster I)	Forward	ATGCAAGTCGAGCGAKG
	Reverse	TATGCGGTATTAATCTYCCTTT
Clostridium leptum (cluster IV)	Forward	GCACAAGCAGTGGAGT
	Reverse	CTTCCTCCGTTTGTCAA
Clostridium perfringens (cluster XI)	Forward	ACGCTACTTGAGGAGGA
	Reverse	GAGCCGTAGCCTTTCACT
Clostridium coccoides (cluster XIV)	Forward	ACTCCTACGGGAGGCAGC
	Reverse	GCTTCTTAGTCARGTACCG
Faecalibacterium prausnitzii	Forward	AACCTTACCAAGTCTTGACATC
	Reverse	TTGCGTAGTAACTGACCATAAG
Lactobacillus spp.	Forward	GAGGCAGCAGTAGGGAATCTTC
	Reverse	GGCCAGTTACTACCTCTATCCTTCTTC
Roseburia spp.	Forward	TACTGCATTGGAAACTGTCG
	Reverse	CGGCACCGAAGAGCAAT
**Bacteroidetes:**		
Bacteroides/Prevotella	Forward	TCCTACGGGAGGCAGCAGT
	Reverse	CAATCGGAGTTCTTCGTG
**Actinobacteria:**		
Bifidobacterium spp.	Forward	CGCGTCYGGTGTGAAAG
	Reverse	CCCCACATCCAGCATCCA
Collinsella aerofaciens	Forward	CCCGACGGGAGGGGAT
	Reverse	CTTCTGCAGGTACAGTCTTGAC
**Archaea:**		
Methanobrevibacter	Forward	CTCACCGTCAGAATCGTTCCAGTC
	Reverse	ACTTGAGATCGGGAGAGGTTAGAGG
**Proteobacteria:**		
Enterobacteriaceae	Forward	CATTGACGTTACCCGCAGAAGC
	Reverse	CTCTACGAGACTCAAGCTTGC
**Verrucomicrobia:**		
Akkermansia muciniphila	Forward	TCTTCGGAGGCGTTACACAG
	Reverse	AGTTGATCTGGGCAGTCTCG

Table S3: Primer sequences for RT-PCR

Gene	Primers (5'→3')	
**Proximal Colon**		
MMP2	Forward	CCTGAATACTTTCTATGGCTGC
	Reverse	GTATGTAGTGGAGCACCAGAGC
MMP9	Forward	GCAACGGAGACGGCAAACC
	Reverse	GACGAAGGGGAAGACGCA
MUC2	Forward	CCACCATTACCACCACCTCAG
	Reverse	CGATCACCACCATTGCCATTG
GPR41	Forward	TCCTCAGCACCCTCAACTCT
	Reverse	CTAGCTCGGACACTCCTTGG
GPR43	Forward	CCGTGCAGTACAAGCTCTCC
	Reverse	CTGCTCAGTCGTGTTCAAGTATT
β-Actin	Forward	TATCGGCAATGAGCGGTTCC
	Reverse	AGCACTGTGTTGGLATAGAGG
**Jejunum**		
ZO-1	Forward	GAGTTTCGGGTCCGAGGAG
	Reverse	CATTGCTGTGCTCTTAGCGG
Occludin	Forward	GAGGACTGGCTCAGGGAATATC
	Reverse	TTGTTGACCTCGTCGAGTTCTG

Table S4: Male and female body composition

	Control	3’SL	2’FL	3’SL+2’FL	p-value
**Males**					
**Total Weight (g)**	464.2± 11.2	426.2±11.5	439.1± 10.1	442.6±11.5	0.14
**Lean+ BMC (g)**	386.9±7.4	366.0±11.8	372.6±8.9	375.9±9.5	0.48
**Fat Mass (g)**	77.3±5.0	63.7±2.9	66.5±3.8	66.7±3.9	0.11
**% Body Fat**	16.5±0.8	15.9±0.8	15.1±0.7	15.0±0.7	0.46
**Bone Mineral Content (g)**	11.0±0.3	10.0±0.3	10.2±0.2	10.5±0.3	0.07
**Bone Mineral Density (g/cm^2)**	0.145±0.002	0.141±0.002	0.143±0.001	0.143±0.002	0.18
**Females**					
**Total Weight (g)**	256.0±4.8	264.1±10.1	269.7±8.8	269.9±8.1	0.59
**Lean+ BMC (g)**	224.8±5.0	226.9±9.4	235.4±5.7	235.2±6.5	0.60
**Fat Mass (g)**	31.2±2.7	37.3±4.0	34.8±3.2	33.8±3.7	0.65
**% Body Fat**	11.5±0.9	14.1±1.4	12.3±0.9	12.8±1.0	0.43
**Bone Mineral Content (g)**	7.2±0.2	7.7±0.3	7.9±0.2	7.9±0.3	0.17
**Bone Mineral Density (g/cm^2)**	0.138±0.002	0.139±0.001	0.141±0.001	0.143±0.001	0.09

Values are means ± SEM, *n* = 8-10. In the overall model, there was a significant sex effect (p=0.0001 for Lean+BMC, Fat Mass, % Body Fat and BMC; p=0.02 for BMD) therefore males and females were analyzed separately. Control: AIN-93; 3’SL: AIN-93 + 3’Sialyllactose; 2’FL: AIN-93 + 2’Fucosyllactose; 3’SL + 2’FL: AIN-93 + 3’Sialyllactose + 2’Fucosyllactose. Significance was set at p<0.05.

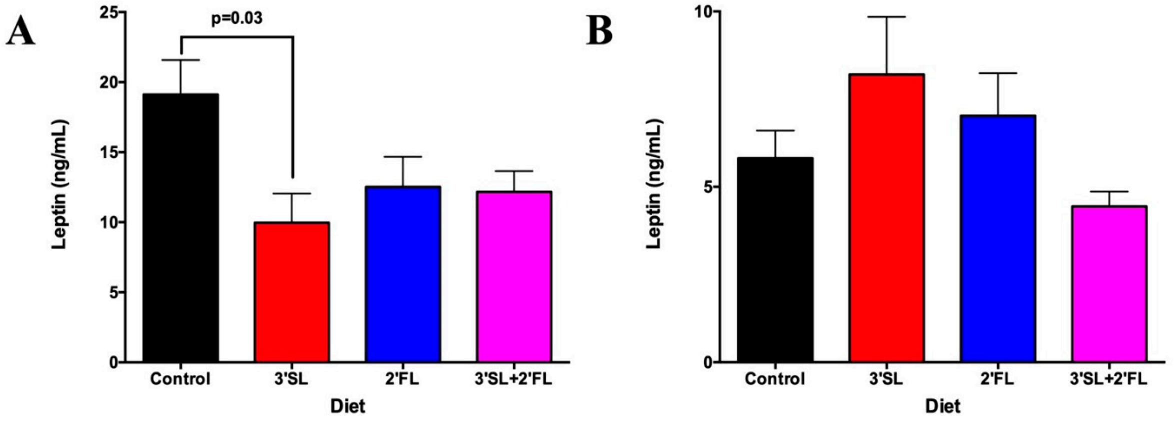

Figure S1: Serum fasting leptin levels at 11 weeks of age in (A) male and (B) female rats fed AIN-93 diet fortified with 3'SL, 2'FL, both or neither for 8 weeks. Values are means ± SEM, *n* = 8-10. In the overall model, there was a significant sex effect (p=0.0001), therefore males and females were analyzed separately. Control: AIN-93; 3'SL: AIN-93 + 3'Sialyllactose; 2'FL: AIN-93 + 2'Fucosyllactose; 3'SL + 2'FL: AIN-93 + 3'Sialyllactose + 2'Fucosyllactose.

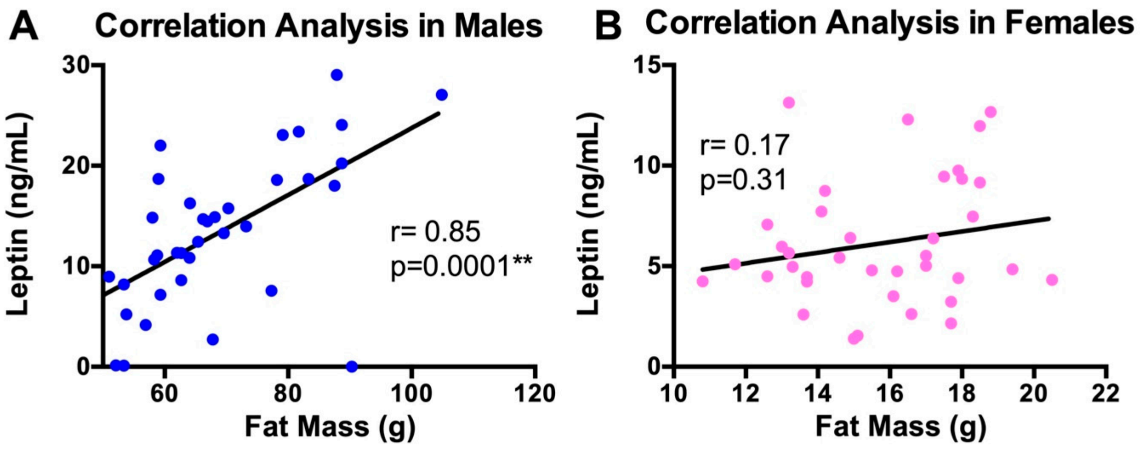

Figure S2: Correlation between fat mass and leptin using a Pearson correlation analysis at 11 weeks of age in (A) male and (B) female rats fed AIN-93 diet fortified with 3'SL, 2'FL, both or neither for 8 weeks. *n* = 38 males, *n* = 38 females.

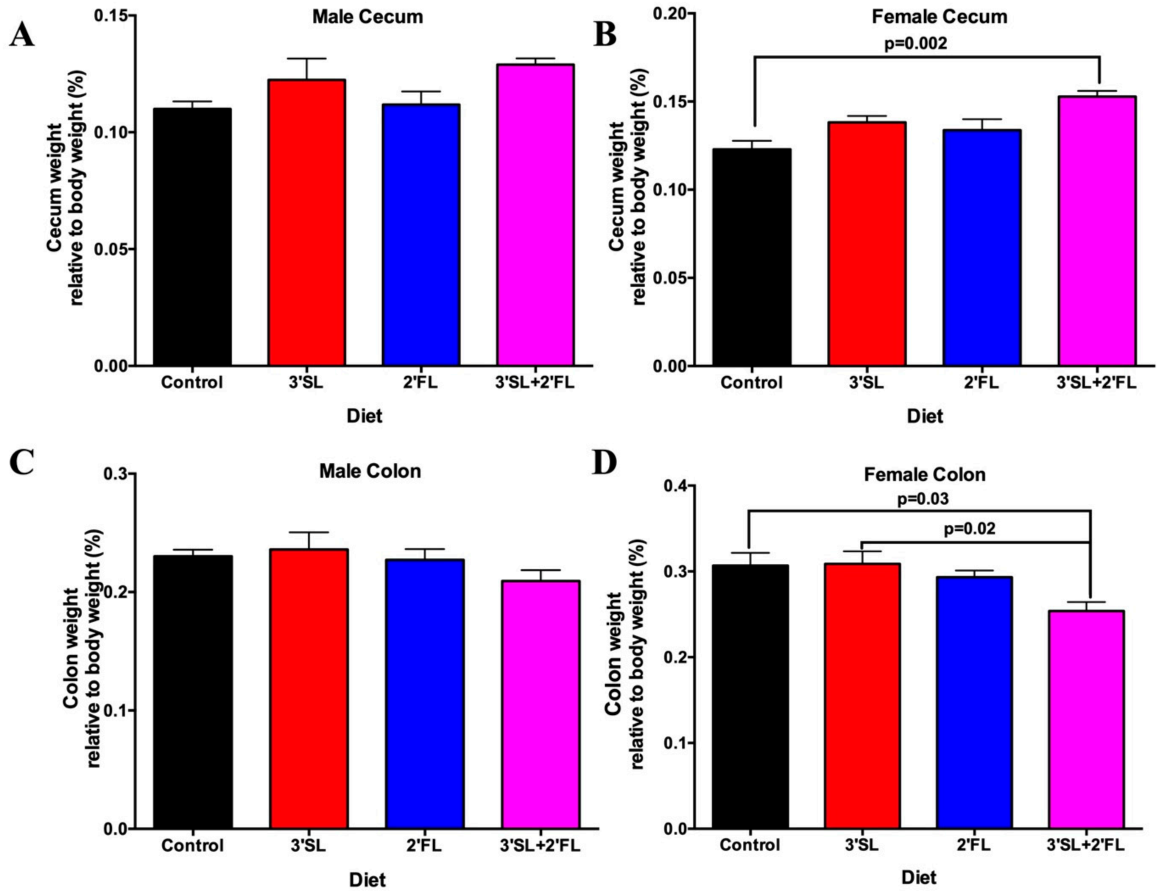

Figure S3: Cecum and colon weight relative to body weight respectively in (A, C) male and (B, D) female rats fed AIN-93 diet fortified with 3’SL, 2’FL, both or neither for 8 weeks. Values are means ± SEM, *n* = 8-10. In the overall model, there was a significant sex effect for cecum and colon (p=0.0001), therefore males and females were analyzed separately. Control: AIN-93; 3’SL: AIN-93 + 3’Sialyllactose; 2’FL: AIN-93 + 2’Fucosyllactose; 3’SL + 2’FL: AIN-93 + 3’Sialyllactose + 2’Fucosyllactose.

Table S5: Male and female serum inflammatory cytokines at 11 weeks of age in rats fed AIN-93 diet fortified diet with 3’SL, 2’FL, both or neither for 8 weeks.

	Control	3’SL	2’FL	3’SL+2’FL	p-value
**Males**					
**TNF *α***	15.4±2.7	13.0±2.2	8.1±1.2	10.4±0.9	0.06
**IL-1α**	43.1±11.5	32.4±7.2	38.4±7.9	41.1±7.8	0.86
**IL-1β**	44.5±5.7	53.5±9.0	36.4±5.4	59.8±11.1	0.22
**IL-5**	113.9±2.2	90.5±11.1	90.4±8.3	85.4±4.9	0.08
**IL-10**	83.3±16.0	64.9±12.6	81.6±16.6	107.7±23.0	0.42
**IL-18**	539.6±81.8[a]	428.1±62.9[ab]	258.0±29.8[b]	263.6±20.3[b]	0.001
**Females**					
**TNF *α***	10.8±1.7	6.0±1.6	8.5±1.6	8.7±1.3	0.23
**IL-1α**	34.4±4.7	33.8±4.7	41.2±10.6	39.1±7.7	0.87
**IL-1β**	48.9±10.1	36.8±5.8	57.4±10.6	40.3±5.2	0.31
**IL-5**	87.4±6.9	85.3±5.1	83.55±8.0	81.5±8.5	0.95
**IL-10**	48.5±8.8	52.8±8.0	84.9±19.0	40.3±7.7	0.07
**IL-18**	220.8±21.4	239.3±19.4	230.4±26.4	202.1±22.3	0.69

Values are means ± SEM, *n* = 8-10. In the overall model, there was a significant sex effect for TNF*α* (p=0.01), IL-5 (p=0.001) and IL-18 (p=0.0001) and a trend for IL-1β (p=0.06) and IL-10 (p=0.07), therefore males and females were analyzed separately. Within males and females, the superscripts[a,b] are used to depict differences between groups where groups without a common superscript differ (P<0.05). Control: AIN-93; 3’SL: AIN-93 + 3’Sialyllactose; 2’FL: AIN-93 + 2’Fucosyllactose; 3’SL+2’FL: AIN-93 + 3’Sialyllactose + 2’Fucosyllactose. All units are pg/mL.

Table S6 Correlation analysis in males between a panel of inflammatory cytokines and genes that maintain intestinal barrier function

	MMP2	MMP9	MUC2	GPR41	GPR43	ZO-1	Occludin
**TNFα**	-0.025	-0.058	0.011	0.248	0.031	0.349	0.219
**IL-1α**	-0.271	0.214	-0.060	0.234	0.038	-0.177	-0.192
**IL-1β**	-0.054	-0.066	0.020	0.057	-0.130	-0.188	-0.014
**IL-5**	0.148	0.244	-0.160	0.188	-0.263	0.183	0.256
**IL-10**	-0.291	0.038	-0.200	**0.368***	-0.289	-0.020	0.082
**IL-18**	0.256	-0.036	-0.203	0.220	-0.190	**0.520****	**0.499****

MMP2, Matrix Metallopeptidase 2; MMP9, Matrix Metallopeptidase 9; MUC2, Mucin 2; GPR41, G Protein-Coupled Receptor 41; GPR43, G Protein-Coupled Receptor 43; ZO-1, Zonula occludens. *p<0.05, **p<0.01.

Table S7 Correlation analysis in females between a panel of inflammatory cytokines and mRNA expression of genes that maintain intestinal barrier function

	MMP2	MMP9	MUC2	GPR41	GPR43	ZO-1	Occludin
**TNFα**	-0.103	-0.018	-0.092	-0.183	-0.033	0.252	0.296
**IL-1α**	-0.114	0.003	-0.177	0.064	-0.103	-0.002	0.186
**IL-1β**	0.172	-0.163	-0.003	0.137	0.056	-0.197	0.096
**IL-5**	0.118	0.025	0.227	0.137	0.070	0.028	0.271
**IL-10**	0.151	-0.125	0.048	0.178	0.141	-0.183	0.114
**IL-18**	0.123	-0.092	**0.358***	0.249	0.039	0.014	0.055

MMP2, Matrix Metallopeptidase 2; MMP9, Matrix Metallopeptidase 9; MUC2, Mucin 2; GPR41, G Protein-Coupled Receptor 41; GPR43, G Protein-Coupled Receptor 43; ZO-1, Zonula occludens. *p<0.05, **p<0.01.

Table S8: Relative abundance of fecal microbiota (qPCR) in male rats at 11 weeks of age fed AIN-93 diet fortified with 3'SL, 2'SL, both or neither

	**Control**	**3'SL**	**2'FL**	**3'SL+2'FL**	**p-value**
			**Relative abundance (%)**		
***Bacteroides/Prevotella* spp.**	2.4±0.7	2.6±0.6	2.4±0.4	3.0±0.5	0.88
***Bifidobacterium* spp.**	0.5±0.1	0.2±0.05	0.3±0.07	0.4±0.1	0.19
Enterobacteriaceae	0.4±0.1[a]	0.2±0.05[ab]	0.1±0.04[b]	0.2±0.04[ab]	0.02
***Lactobacillus* spp.**	44.9±7.5	57.9±12.5	33.3±11.0	46.9±10.0	0.48
***Clostridium perfringens* (cluster I)**	1.4±0.3	0.7±0.2	1.0±0.2	0.7±0.1	0.11
***Clostridium leptum* (cluster IV)**	14.3±2.2	6.9±1.4	12.0±3.2	8.5±1.9	0.12
***Clostridium difficile* (cluster XI)**	0.34±0.07	0.4±0.06	0.2±0.01	0.2±0.03	0.06
***Clostridium coccoides* (cluster XIV)**	31.1±5.5	23.3±4.2	24.3±4.4	22.3±2.9	0.48
***Roseburia* spp.**	0.002±0.0002[a]	0.01±0.005[b]	0.002±0.0003[a]	0.002±0.0004[a]	0.01
***Methanobrevibacter* spp.**	0.01±0.002	0.02±0.003	0.02±0.003	0.02±0.002	0.53
Akkermansia muciniphila	0.2±0.07	0.04±0.03	0.08±0.03	0.01±0.006	0.14
Faecalibacterium prausnitzii	0.1±0.03	0.07±0.02	0.07±0.01	0.1±0.03	0.08
Collinsella aerofaciens	0.02±0.002	0.01±0.002	0.01±0.003	0.02±0.004	0.12
**Total bacteria (16S rRNA gene copies)**	39,654,166±6,540,301	41,778,495±5,162,862	42,574,434±6,551,598	44,569,545±2,780,744	0.93

Values are means ± SEM, *n* = 8-10. *Methanobrevibacter* spp., *Akkermansia muciniphila, Faecalibacterium prausnitzii* and *Collinsella aerofaciens* were log transformed. Total bacteria are represented as 16S rRNA gene copies/20 ng genomic DNA. All other taxa are presented as relative abundance (%) of bacterial taxa per total bacteria (16S rRNA gene copies / total 16S rRNA gene copies). 3'SL: AIN-93 + 3'Sialyllactose; 2'FL: AIN-93 + 2'Fucosyllactose; 3'SL+2'FL: AIN-93+ 3'Sialyllactose + 2'Fucosyllactose. The superscripts[a, b] are used to depict differences between groups where groups without a common superscript differ (*P*<0.05).

Table S9: Relative abundance of fecal microbiota (qPCR) in female rats at 11 weeks of age fed AIN diet fortified with 3'SL, 2'SL, both or neither

Treatment	Control	3'SL	2'FL	3'SL+2'FL	p-value
			Relative abundance (%)		
***Bacteroides/Prevotella* spp.**	3.3±0.7	3.0±0.4	1.5±0.3	2.4±0.5	0.07
***Bifidobacterium* spp.**	0.2±0.1	0.1±0.01	0.2±0.04	0.2±0.04	0.28
**Enterobacteriaceae**	0.3±0.1	0.2±0.05	0.3±0.1	0.2±0.03	0.42
***Lactobacillus* spp.**	21.9±7.5	34.8±14.6	26.0±9.5	24.4±10.1	0.85
***Clostridium perfringens* (cluster I)**	1.2±0.2[ab]	0.4±0.1[a]	1.6±0.4[b]	0.6±0.1[a]	0.002
***Clostridium leptum* (cluster IV)**	14.4±1.8	8.8±1.5	8.5±1.8	13.9±2.6	0.19
***Clostridium difficile* (cluster XI)**	0.4±0.1	0.3±0.04	0.3±0.04	0.3±0.04	0.24
***Clostridium coccoides* (cluster XIV)**	30.7±3.1	26.5±4.6	24.5±5.6	16.0±3.7	0.15
***Roseburia* spp.**	0.006±0.002	0.002±0.0004	0.002±0.0004	0.002±0.0003	0.07
***Methanobrevibacter* spp.**	0.02±0.003[ab]	0.03±0.005[a]	0.01±0.002[b]	0.02±0.003[b]	0.002
Akkermansia muciniphila	0.2±0.07[a]	0.02±0.007[ab]	0.07±0.03[ab]	0.007±0.003[b]	0.009
Faecalibacterium prausnitzii	0.1±0.03	0.07±0.03	0.1±0.01	0.1±0.02	0.46
Collinsella aerofaciens	0.02±0.003	0.02±0.003	0.02±0.004	0.02±0.003	0.10
**Total bacteria (16S rRNA gene copies)**	32,776,158±2,447,995[ab]	23,426,482±903,946[ab]	39,659,548±6,931,952[a]	20,657,973±2,909,108[b]	0.02

Values are means ± SEM, *n* = 8-10. *Bacteroides* spp.*, C. leptum, Roseburia* spp.*, A. muciniphila* and *C. aerofaciens* were log transformed. Total bacteria are represented as 16S rRNA gene copies/20 ng genomic DNA. All other taxa are presented as relative abundance (%) of bacterial taxa per total bacteria (16S rRNA gene copies / total 16S rRNA gene copies).3'SL: AIN-93+ 3'Sialyllactose; 2'FL: AIN-93 + 2'Fucosyllactose; 3'SL+2'FL: AIN-93 + 3'Sialyllactose + 2'Fucosyllactose. The superscripts[a, b] are used to depict differences between groups where groups without a common superscript differ (*P*<0.05).

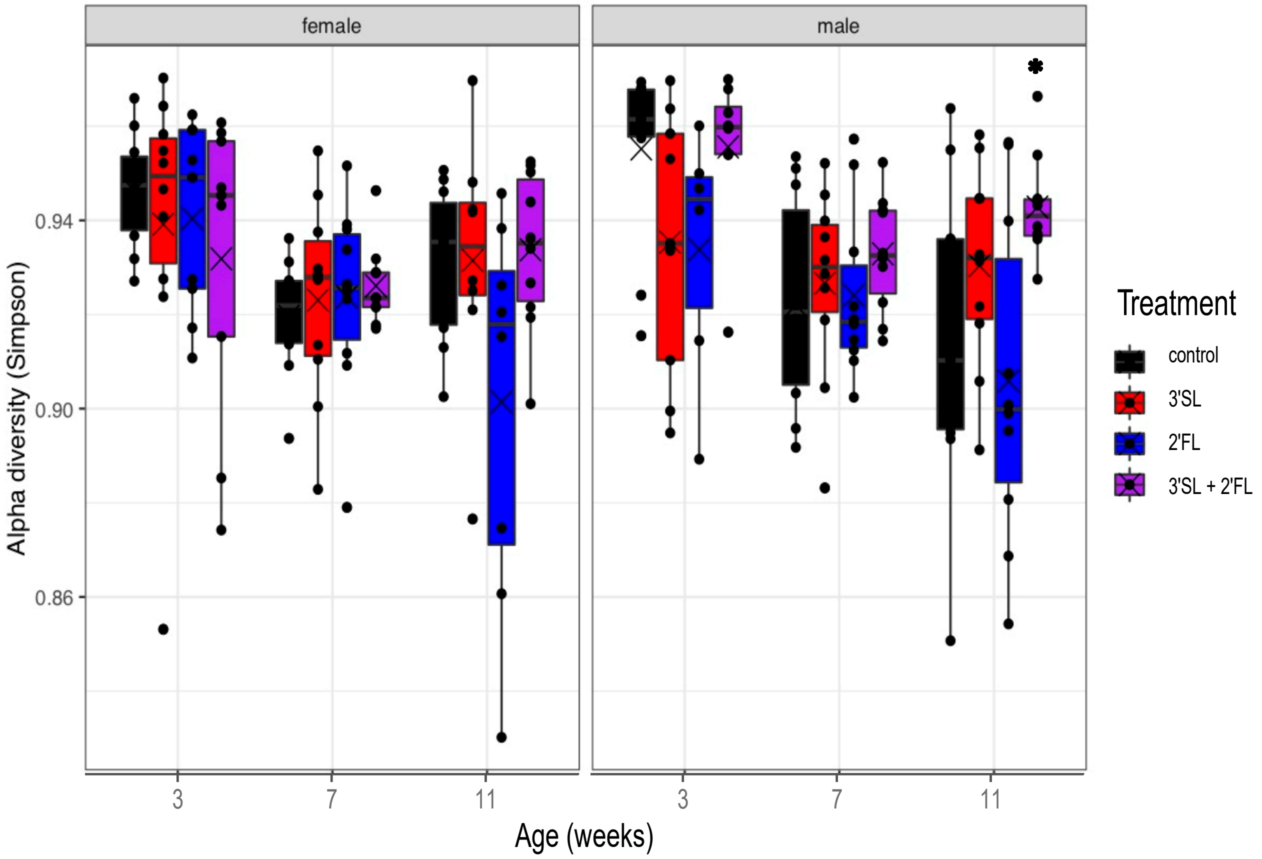

Figure S4: Male and female alpha diversity according to the Simpson index using the phyloseq package at 3, 7 and 11 weeks of age. Control: AIN-93; 3’SL: AIN-93 +3’Sialyllactose; 2’FL: AIN-93 + 2’Fucosyllactose; 3’SL + 2’FL: AIN-93 + 3’Sialyllactose + 2’Fucosyllactose. *Significance was set at $p<0.05$.

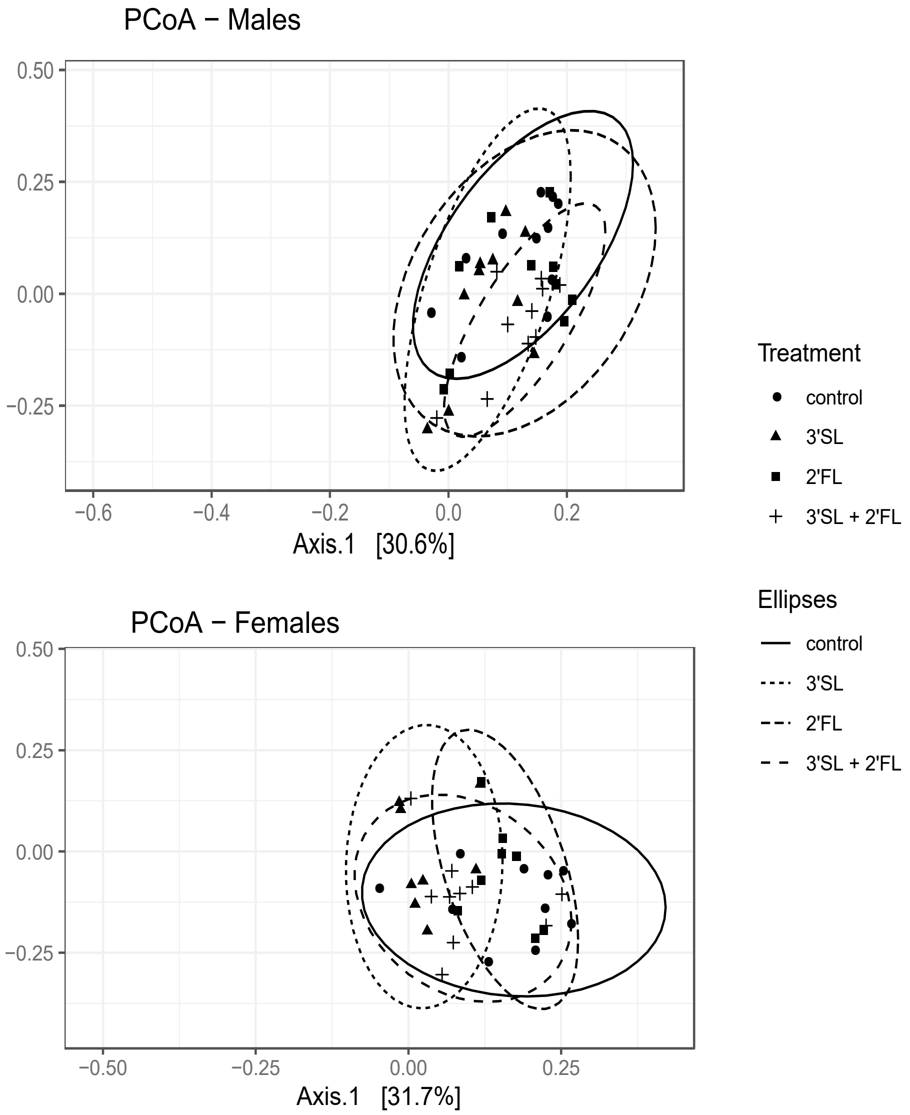

Figure S5: Male and female beta diversity at 11 weeks of age calculated with principle coordinates analysis (PCoA) using a Bray-Curtis distance matrix. ASVs are normalized proportionally, by relative abundance. ASVs present in less than 5% of the samples were removed. Control: AIN-93; 3’SL: AIN-93 +3’Sialyllactose; 2’FL: AIN-93 + 2’Fucosyllactose; 3’SL + 2’FL: AIN-93 + 3’Sialyllactose + 2’Fucosyllactose.

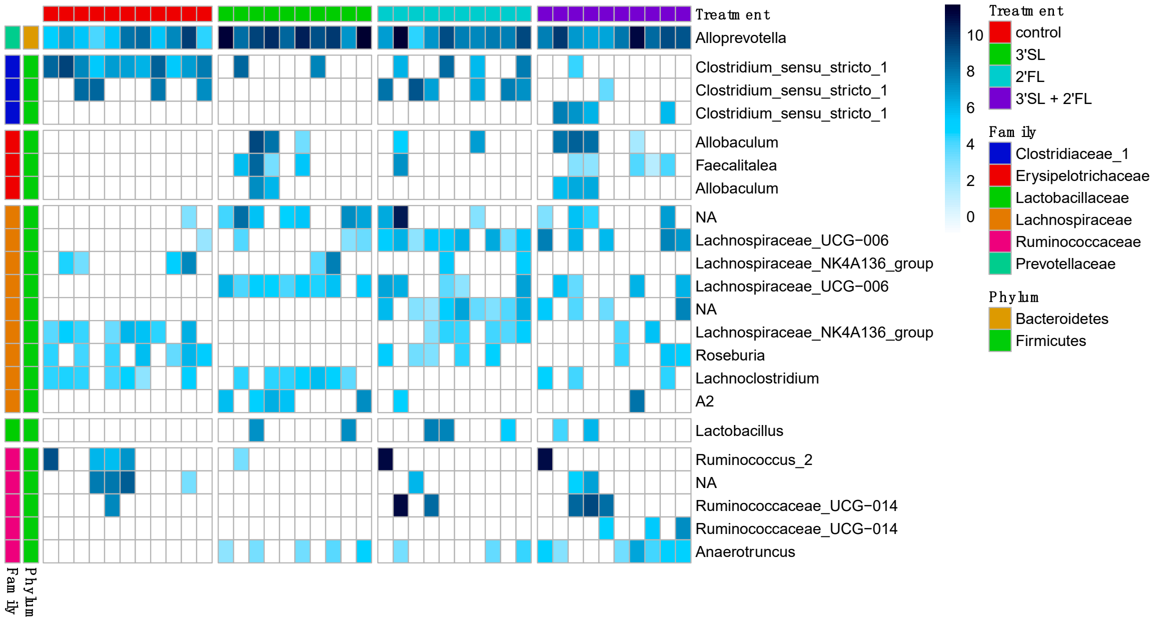

Figure S6: Heatmap of top gut bacterial phyla, families and genera between treatments in males at 11 weeks of age. Control: AIN-93; 3'SL: AIN-93 +3'Sialyllactose; 2'FL: AIN-93 + 2'Fucosyllactose; 3'SL + 2'FL: AIN-93 + 3'Sialyllactose + 2'Fucosyllactose.

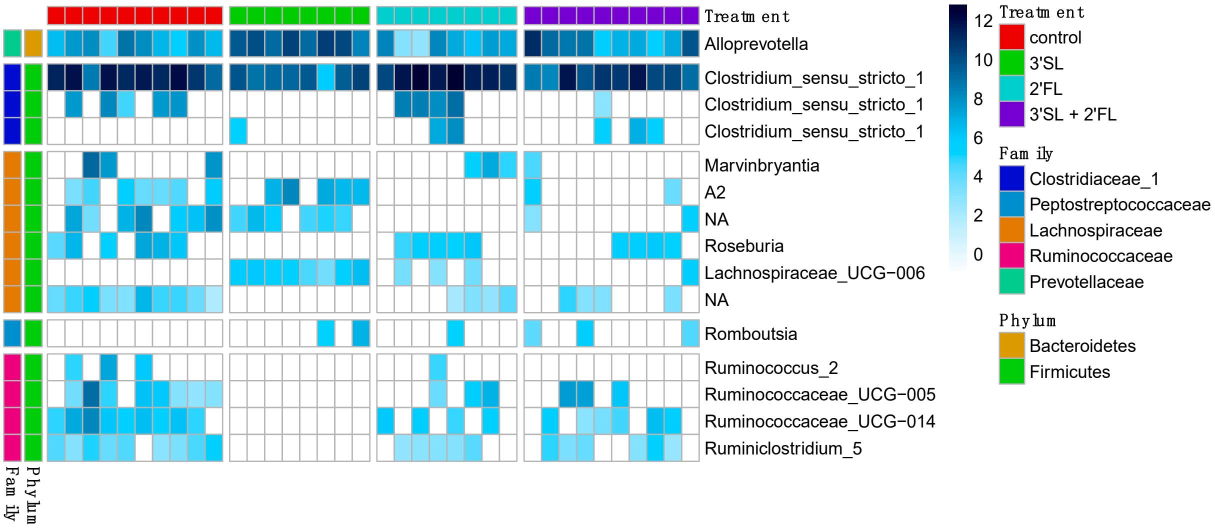

Figure S7: Heatmap of top gut bacterial phyla, families and genera between treatments in females at 11 weeks of age. Control: AIN-93; 3'SL: AIN-93 +3'Sialyllactose; 2'FL: AIN-93 + 2'Fucosyllactose; 3'SL + 2'FL: AIN-93 + 3'Sialyllactose + 2'Fucosyllactose.
